# Experimental development based on mapping rule between requirements analysis model and web framework specific design model

**DOI:** 10.1186/2193-1801-2-123

**Published:** 2013-03-21

**Authors:** Hirotaka Okuda, Shinpei Ogata, Saeko Matsuura

**Affiliations:** 1 Division of Electrical Engineering and Computer Science, Graduate School of Engineering and Science, Shibaura Institute of Technology, 307 Fukasaku, Minuma-ku, Saitama, Saitama, 337-8570 Japan; 2 Department of Computer Science and Engineering, Faculty of Engineering, Shinshu University, 4-17-1 Wakasato, Nagano, Nagano, 380-8553 Japan

**Keywords:** Web frameworks, Unified modeling language, Requirements analysis model

## Abstract

Model Driven Development is a promising approach to develop high quality software systems. We have proposed a method of model-driven requirements analysis using Unified Modeling Language (UML). The main feature of our method is to automatically generate a Web user interface prototype from UML requirements analysis model so that we can confirm validity of input/output data for each page and page transition on the system by directly operating the prototype. We proposes a mapping rule in which design information independent of each web application framework implementation is defined based on the requirements analysis model, so as to improve the traceability to the final product from the valid requirements analysis model. This paper discusses the result of applying our method to the development of a Group Work Support System that is currently running in our department.

## Introduction

Nowadays many useful services and products as computer systems requires the developers to efficiently develop high quality systems, so that they can meet a diversity of users with different knowledge, a diversity of use cases, and a diversity of such input/output devices as smart phones and tablet PCs. To ensure such development, it is important that software has traceability through the life cycle, and Model Driven Development (Mellor et al. 2002) is a promising approach.

We have proposed a method of model-driven requirements analysis (Ogata & Matsuura 2008; 2010a) using Unified Modeling Language (UML). The main feature of our method is to automatically generate a Web user interface prototype from UML requirements analysis model so that we can confirm validity of input/output data for each page and page transition on the system by directly operating the prototype. We have shown that the requirements analysis model has traceability to the final product by implementation experiment from the requirements analysis model (Ogata & Matsuura 2010b), but such systematic method as a mapping rule between requirements analysis model and the design model remains to be provided.

Recently, most of web applications are developed by using such existent web application frameworks as struts, play frameworks, etc., a web application framework is a set of reusable web application specific architecture classes. Defining application specific concrete class by the developers leads to the final web application source codes. Therefore the developers are required to know the web application specific architectural mechanism of each concrete framework such as struts, etc. Such concrete knowledge dependent to each framework implementation seems to disturb the efficient high quality development.

This paper proposes a mapping rule in which design information independent of each web application framework implementation is defined based on the requirements analysis model and it can automatically generates each framework specific codes.

## Requirements analysis method

### Modeling features of requirements analysis method

At requirements analysis phase, developers extract requirements for a system from customers and generally specify them by defining semiformal documents. Recently, many developers have been getting to use UML, so that requirements specifications can be defined more formally. We have proposed a method of model-driven requirements analysis using UML. We analyze functional requirements of services as well as use case analysis. Especially, because what customers essentially want to do obviously appear within the interaction between a user and a system, our method proposes to clearly model the interaction.

To put it concretely, we specify business process as a service from the following four viewpoints.

- Based on the business rules, what kinds of input data and the conditions are required in order to execute a service correctly?
- To observe the business rule, what kinds of conditions should be required in case of not executing the service? Moreover, how the system should treat these exceptional cases?
- According to these conditions, what kinds of behaviors are required in order to execute the service?
- What kinds of data are outputted by these behaviors?

Based on the above mentioned four viewpoints, both business flow and business entity data which are required to execute the target business are defined by activity diagrams and a class diagram in UML.

An activity diagram specifies not only normal and exceptional action flows but also data flows which are related with these actions. An action is defined by an action node and data is defined by an object node being classified by a class which is defined in a class diagram. Accordingly, these two kinds of diagrams enable us to specify business flow in connection with the data. This is one of the features of our method on how to use activity diagram and class diagram. Especially, the interaction between a user and a system includes requisite various flows and data on user input, conditions, output to execute a service correctly.

The second feature is that an activity diagram has three kinds of partitions being named *User*, *Interaction*, and *System*. This is because that these partitions enable us to easily recognize the following activities; user input activities, interaction activities between a user and a system which are caused by the conditions to execute a service, and the resulted output.

The third feature is that we use an object diagram to define concrete data for each activity, because concrete valid data make it easy for us to confirm business process.

The fourth feature is that a prototype which consists of Web pages written in HTML is automatically generated from these three kinds of diagrams. The prototype which is a kind of final product model enables the customers to confirm plainly and easily the requisite business flows in connection with the data. The generated prototype describes the required target system except user interface appearance and internal business logic processing. Moreover, the prototype enables the developer to confirm and understand the correspondence between his/her models and the final system. The developer defines three kinds of diagrams along requirements analysis from such different viewpoints as action flows, data flows and the structure, and the concrete values. The automatically generated prototype enables him/her to easily understand the consistency between his/her models and the target system. To be able to fully understand the correspondence between each diagram and the target system, a prototype can be generated whenever the developer want to confirm at the requirement analysis phase. The requirement analysis model is defined by using the (astah*, http://www.change-vision.com/) of a modeling tool.

At the stage where the customers have confirmed that the prototype satisfactorily represents their requirements, we can say that the customers can validate that the specification meets their expectations from the viewpoint of the actual usage.

However, it is important that the developer can verify the specification so as to confirm the feasibility of the specification. To do this, the developers must confirm that a sequence of actions and data flows within the system partition of the activity diagrams can produce the expected output data from the specified input data. The system side prototype helps the developers to confirm the following facts.

- Input data being defined by the user can be transformed into entity data of the system.
- The existent entity data that should be generated via the other use cases and the above mentioned entity data can generate the target output data following the specified action sequence.

As a result of consideration like this, the developers can define entity classes.

### Specified components of requirements analysis model

After requirements analysis using our method, we can get the following specified components.

1. Entity Classes: A set of entity classes which are within the system partition of all activity diagrams becomes candidates of persistent objects.
2. Input/Output Data Classes: A set of input/output data classes which are within the user or interaction partition of each activity diagram decides a both a sequence of pages of the service and the structure of each page.
3. Trigger and Process Flow: Each activity diagram decides a sequence of process flow of the service. A process flow is a sequence of action nodes and object nodes within the system partition which is called by executing a trigger message in an input class.
4. Exceptional Flow: When an exception for the inputted data is happened, an activity diagram decides a process flow of the system.

## Problems in web application development

Many current web applications give us richer client service by such technology as CSS, Flash, Java Script, and Ajax, etc. Server side services also have been developing through such technology as PHP, JSP, ASP.NET, CGI, etc.. Moreover, a data persistence mechanism has been developing through such as DBMS, RDBMS, etc.. Web application framework aims to alleviate the overhead associated with common activities by providing libraries for templating framework, database access, security management, etc. As a result, we can focus on designing the application logic independent of the web application concerns. Most web application consists of client-side, server-side and persistence-side that the so-called ‘Model-View-Controller’ pattern which can improve the mutual independency.

However, different framework has different libraries, so that the developers are obliged to implement a system in individually different expressions. Moreover, the usage depends on the intention of developers, so that sometimes they define unclear implementation of MVC model which will influence the maintainability. Especially they sometimes fail in giving appropriate roles to the interaction between a user and the system for lack of clear guideline. To improve the maintainability of frequently changeable web application, it is required that a clear mapping rule forms a relation between requirements component and hot spots of a framework.

We propose a mapping rule between the above mentioned components, which have clarified guideline for the interaction by our requirements analysis method, a web framework specified design model, which is independent of individual implementation.

## Mapping rule between requirements analysis model and Web framework specified model

We define a relation between requirements component and hot spots of a web framework and describe how to design these hot spots according to the components.

### Web framework specified design model

**Figure 1:**
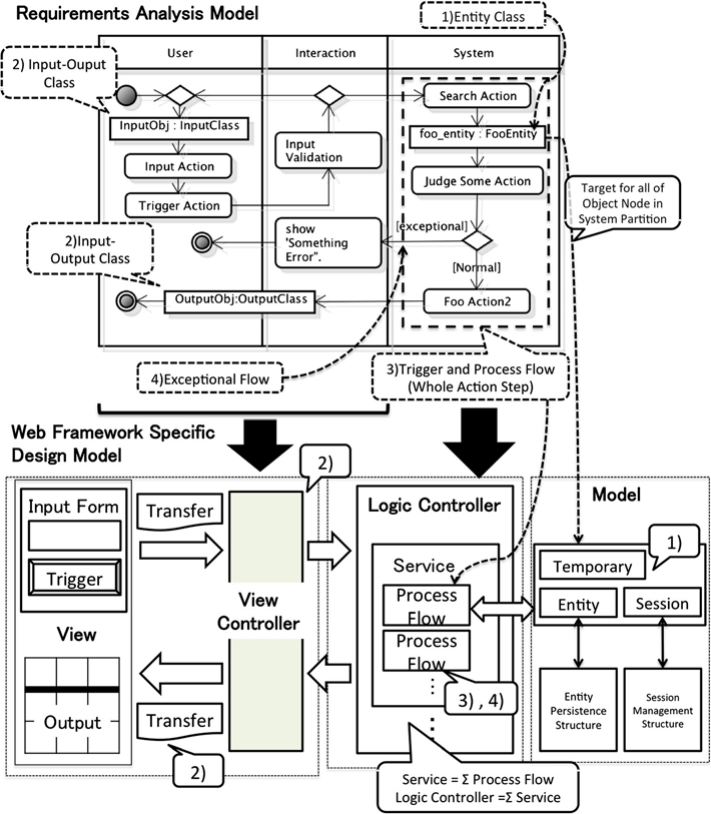
**Requirements Analysis Model and Web Framework Specific Design Model.**

Figure 1 shows a Web Framework Specific Design Model that is specified in accordance with a Requirement Analysis Model. It consists of four roles such as View, View Controller, Logic Controller, and Model. View embodies each page in accordance with the input/output data requirements analyzed in the user and interaction partition. View Controller embodies such four roles as validating of input data, transferring input data to Logic Controller, decomposing transferred object to output data in View Controller, controlling a process called by a trigger. Logic Controller embodies control requirements in the system partition, which is a process flow called by a trigger. It is a sequence of methods which are embodied actions in the system partition. Model embodies the entity classes in the system partition. As Logic Controller and Model are independent of framework implementation, they can become reusable components. On the other hand, View and View Controller should specify the above-mentioned roles with rules of each framework.

Additionally, we need to design that the persistent data can keep the consistency during the execution, so that all services can be used by multiple concurrent users at the same time without the inconsistency. A set of entity classes which are within the system partition of all activity diagrams becomes candidates of persistent objects. At this point of view, we have to specify the role of each object within the system partition in an activity diagram. In an activity diagram, an object node represents an instance data of a class that is valid within several particular scopes. The scopes are classified by both the processing unit in Process Flow and an executing process with multiple concurrent users as follows.

- Persistence for DB: Persistence data that will be shared by multiple concurrent users needs to be stored in the database.
- Session by State: Session data needs identifying within an executing process of a single user, so as to keep the consistency between the others.
- Session by Page Transition: Session data needs passing the identifying individual data to the other Process Flows during a service so as to keep the consistency within the single user execution.
- Temporary: Temporary Data is valid within only a Process Flow. It holds input/ output data of the Process Flow by GET/POST function.

### Components of web framework specific design models

**Figure 2:**
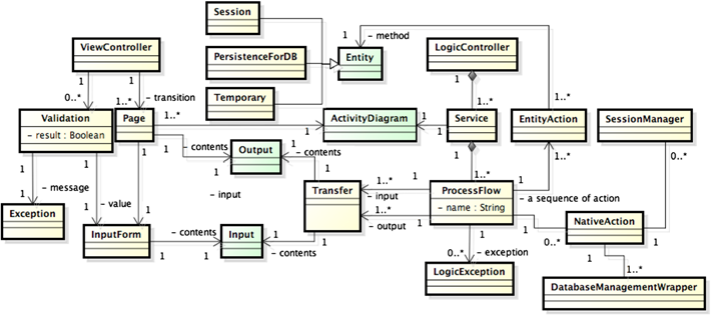
**ViewController and LogicController.**

Figure 2 shows a meta model of both View Controller and Logic Controller.

A process flow in the requirements analysis model has a corresponding name of a trigger in input class and consists of a sequence of several entity actions. Every action on the system partition is allocated as a business logic method to an appropriate entity class, that is, an entity action. An objective word of the action description is a hint for allocating to an entity class. A process flow also has several logical exception s handling in accordance with the business rules. The number of LogicException objects is the same number of exceptional flows in the activity diagram of the requirements analysis model.

To make a relation between view controller and logic controller, data transfer classes are generated from input/output data classes shown in the requirements analysis model. Transfer Class is defined by removing some trigger message of input/output data class and its field should be specified by primitive type and string type so as not to depend on any framework, That is, it plays a role of a mediator between ViewController and LogicController and each process flow has Transfer object as the input/output data.

Based on the specified entity data, SessionManeger takes a responsibility of managing the above mentioned session data. DatabaseManagement is a class that manages the persistent data stored in the database.

We have proposed a constraint table which represents conditions on input data and the exceptional handling and the message (Ogata & Matsuura Ogata and Matsuura 2011). Based on the constraint table, exception checking action on the interaction partition is specified by some invariant conditions on input data. We call it Validation. Validation is implemented by using an exception handling mechanism of each framework.

## Implementation experiment and discussion

### Outline of development

We have developed a Group Work Support System that aims to support a lesson for Project Based Learning by applying our method. The system is called GWSS. Participants of this lesson are about 25 students and 1 teacher and several teaching assistants. The students are divided into several groups. Each participants have own authority according each role except that a teaching assistant has the same authority with a teacher. The system has the following three use cases to manage tasks during the group work.

- Bulletin Board: It is used at group discussion between group members and Q&A between the students and the teaching members.
- The Minutes of Meeting: It aims at sharing various information on the development with group members and recording the discussion results.
- The Report of Tasks: The intention of the report is that a student plans his/her own tasks on the following week and reports the results.

The authorities are given to the participants as Table 1 shows. GWSS has also the functions to login in the system and to manage the participant student’s information.

**Table 1:** **The authorities for the functions of GWSS**

	Minutes of meeting	Bulletin board	Report of tasks
Student	Write/Read (Own Group Only)	Write/Read (Own Group Only)	Write/Rea (Own Group Only)
Teacher	Read (All Student Groups)	Write/Read (All Student Groups)	Read (All Student Groups)

### Development process and environment

We have conducted implementation experiment by using Play! Framework 1.2.4(Play! Framework, http://www.playframework.org/), MySQL 5.5 and Hibenate implement the database management and the O/R mapping between Model and DB. Two graduate students who have a development experience of a certain scale software system have developed GWSS in three weeks. The number of lines of code is 4946 in total. Table 2 shows the number of components of the RA model and the final source code.

**Table 2:** **The number of components in RA model and source code**

	RA model	Source code
Use case	12	
All actions	252	
All classes	50(1)	65
All attributes	157	98
All methods	0(34)	164
Lines of code		4946

We defined the requirements analysis model of GWSS by using our model driven requirements analysis method. The resultant analyzed model includes 12 use cases which are defined by 12 activity diagrams, 16 entity classes and 33 input/output classes. The average of the number of action nodes in an activity diagram is 21. The average of the number of attributes in a class is 3. The number of classes denoted in parentheses represents added class at defining the Web Framework Specific Design Model. All methods were defined based on actions in the system partition corresponding with the Triggers and Process Flows. The number of methods in the source code increases by the reason that utility and several methods which implement the business logic need defining. The number of attributes decreases by the reason that almost attributes in input/output classes is replaced into the forms in HTML file.

In accordance with the mapping rule, we define the Web Framework Specific Design Model as follows.

First, we specify the role of each object within the system partition in an activity diagram. Secondly, a skeleton code is generated from a sequence of methods corresponding with actions in the Process Flow, that becomes a method called by the Trigger. Thirdly, input/output classes define View, which is written in HTML, so that it includes a trigger which calls the Trigger and the input/output data, which has a role of ViewController.

### Example for BBS implementation

**Figure 3:**
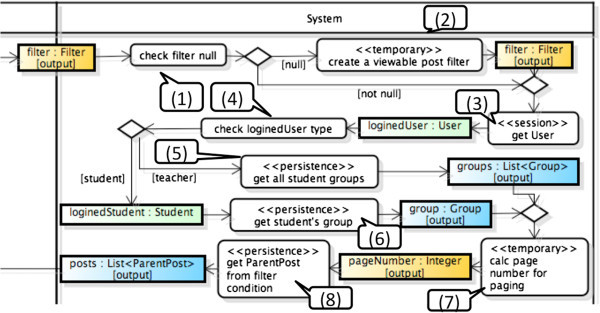
**A portion of activity diagram for browsing BBS.**

**Figure 4:**
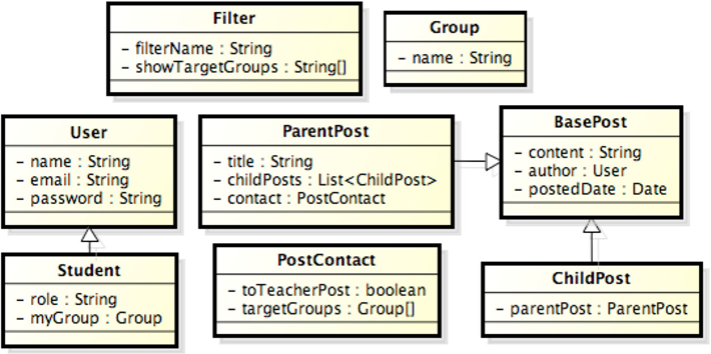
**Classes with persistence for DB on browsing BBS.**

Figure 3 shows a portion of the activity diagram for browsing BBS. Figure 4 shows the classes defined in the system partition of the activity diagram. We specify the role of each object within this diagram as follows.

- *Filter* has a role of Temporary because it is valid within only a Process Flow.
- *User* is Persistence for DB that is created and updated in the other use cases. On the other hand, in this use case, *User* is used to identify the subject of an n executing process. That is, *User* has a role of Session by State in this case.
- Because both *ParentPost* and *Group* are valid across different use cases, they have a role of Persistence for DB.

Moreover, an object node on the boundary line between the interaction partition and the system partition can clearly specify the input data to the Process Flow. Several object nodes denoted by “output” will be used as the output data of the Process Flow.

Next, we will explain how to derive the following source code of Play! Framework from the defined Web Framework Specific Design Model.

A method *viewBBSProcess* expresses the Process Flow described in Figure 3. An object node on the boundary line is a parameter of the method and has a type *Filter*. The return value has a type *Map<String, Object>* because this method returns multiple values which are represented by object nodes denoted by “output”. The source code line 2 expresses the declaration of return value. In the source code line 8, 11, 14, and 15, each value of object node with “output” is stored in the Map. All numbers written in the balloon in Figure 3 correspond to the line number of the source code. The number 7 in Figure 3 expresses an action of paging. We implement the action by a third-party module in play framework.

Play framework provides a specific method index to display a top page to activate BBS. ViewController is defined by this method as shown in the following source code. The source code line 2 expresses a call of Logic Controller. After line 5, all objects are gotten to generate the required output data. After line 20, these data are transformed into another data by Transfer class. Line 22 expresses a specific method of play framework, which has a role to return the requisite data to display BBS. From line 22 to line 26 shows a process of paging by using library functions.

## Discussion

This section discusses a comparison between our previous toy problem (Okuda et al. 2012) and the developed product. There are the following 3 points of view for this method. (1) Transfer, (2) Logic Controller and View Controller, (3) Problems through this development.

### Transfer

In the previous study, Transfer object is defined to pass input/output values between each the controller and the view, so as to reduce the framework dependency to reuse Logic Controller.

In this experiment, using the Transfer object is able to reduce dependence of the View function for reducing framework dependency from the practical viewpoint. The View, which has high functionality such as being able to call logic controller and use *if* statement and loop statement and create any objects, increase the dependency because of difficult to divide responsibility of the View and View Controller.

But a case of what has same thing to show in page and entity field and an object which function is ‘Temporary’, is a disadvantage because of redundant class definition and so increasing complexity for managing source code. In this case, the effects of reusability didn’t appear by using transfer, because of existing few classes over several use cases.

### Logic controller and view controller

A key idea of this method is that creating meta-model by using Façade Pattern. In previous study, Logic Controller is independent from framework element and creates Process Flow from Activity Diagram. In Process Flow, There are multi input and single output and exceptions.

In this experiment, a key idea was effective following chapter 4.3. But the change point is that process flow can return multi output because of the fact of many output (following Figure 3). Constituting Logic Controller and Service by the meta model, it is difficult to manage source code by many files. So we constitute of a include class which include logic controller and view controller.

In Process Flow, Each action in System Partition of Activity Diagram allocate to entity method. In this experiment, there are two pattern of the action. In Process Flow, each action in System Partition of Activity Diagram allocate as entity method. But, in this experiment, there are 3 types of action following facts. (1) An action is integrated other action. (2) An Action has original code of Process Flow, such as ‘*instanceof*’ statement in Java. (3) An Action is mapped clearly for entity method. The disadvantage of allocating entity method is that verbose description in actions step doesn’t collect up. It is difficult for developer to extract common point and utility for logic, because of an action clear only input and output.

### Problems though development

#### First, discussing about model of persistence candidates

All of Object Nodes in System Partition of Activity Diagrams are assumed to use by single user. But it is need to clear where the data is stored to use multi user. So all of object nodes have 4 functions. Actions in System Partition corresponded to source code of persistence mechanism such as JPA (Java Persistence API) and Session through *SessionManager* and entity methods. So process flow is not independent from framework specific elements. In the future, it is necessary to divide Model from Persistence mechanism for software reusability and flexibility.

#### Next, discussing about view and authority

The View is template of viewing surface and generates a page from pouring data from View Controller. In this experiment, View is very complexity because of including two authorities (teacher and student) in a same template file to respond to flexible changes. The View has many consideration point of authority. So we can organize about this point.

#### Finally, discussing about well-known components such as paging and so on

When the application decides to implement web architecture, we want to select from well-known component to create easily the web application. So we can organize well-known component sets for web.

## Related works

Zhou and Chusho (2009) discusses the reusability of domain specific web application framework. They focus on Struts framework from the implementation viewpoint, but this paper applies three frameworks by using requirements analysis method from the implementation independent viewpoint. So our methods provide to be able to design process for developers by using organized design information.

There are domain specific web application development methods by original element and its connections (Ogata & Matsuura 2008; WebML, http://www.webml.org/webml/page1.do). But it is difficult for the developers to learn how to describe. Other hand, our method is described by UML based on common recognition (for example, class diagram and an activity diagram, etc.…). Liu & Li (2010) proposes a new web framework by revealing web application design information. We propose a consistent method from requirements analysis through to implementation, so that we can design from what was requirements ensure the validity.

A requirements analysis model can be verified by user of using the prototype which presents data input/output of providing services and normal operating procedures and exception handlings. Developers define the providing service from the perspective of user and system interaction. The feasibility can be verified from the perspective of relationship between input/output class and entity and entity life-cycle by developer.

Defining model by the above process and adding the required design information, developers can apply the model to any web application frameworks in a systematic way. As a result, the prototype can easily be richer.

## Conclusion and future works

We have been proposing requirements analysis method which make sure of requirements, which has interaction between system and user, through auto generated web prototype. But that method is not clearly designing and implementation process. Furthermore, there are rapidly changing requirements for web application, developer keep maintainability for system.

This paper proposes that high maintainability systematic design methodology for web framework and requirements analysis model called Web Framework Specific Design Model and its mapping rules. This paper indicates the validity for implementing restaurant order management system for two frameworks.

It is our future work that the entity action of Process Flow becomes perfectly independent of the corresponding persistence APIs of source code.
